# Socioeconomic inequalities in adherence to clinical practice guidelines and breast cancer survival: a multicentre population-based study in Spain

**DOI:** 10.1136/bmjqs-2024-017809

**Published:** 2024-12-31

**Authors:** Dafina Petrova, Daniel Redondo-Sánchez, Miguel Rodríguez-Barranco, Rafael Marcos-Gragera, Marcela Guevara, Marià Carulla, Arantza López de Munain, Ana Vizcaíno, Sonia del Barco, Encarnación González-Flores, Marina Pollán, María-José Sánchez

**Affiliations:** 1 Instituto de Investigación Biosanitaria ibs.GRANADA, Granada, Spain; 2 CIBER de Epidemiología y Salud Pública (CIBERESP), Madrid, Spain; 3 Escuela Andaluza de Salud Pública, Granada, Spain; 4 Medical Oncology, Hospital Universitario Virgen de las Nieves, Granada, Spain; 5 Epidemiology Unit and Girona Cancer Registry, Oncology Coordination Plan, Catalan Institute of Oncology (ICO). Girona Biomedical Research Institute (IDIBGI-CERCA), Girona, Spain; 6 Josep Carreras Leukemia Research Institute, Girona, Spain; 7 Department of Medical Sciences, Medical School, University of Girona, Girona, Spain; 8 Registro de Cáncer de Navarra, Instituto de Salud Pública y Laboral de Navarra, Pamplona, Spain; 9 Tarragona Cancer Registry, Hospital Universitari Sant Joan de Reus, Reus, Spain; 10 Basque Country Cancer Registry, Basque Government Department of Health, Vitoria-Gasteiz, Spain; 11 Castellón Cancer Registry, Health Department Generalitat Valenciana, Castellón, Spain; 12 Instituto de Salud Carlos III, Madrid, Spain

**Keywords:** Clinical practice guidelines, Evidence-based medicine, Healthcare quality improvement

## Abstract

**Introduction and aims:**

Women residing in lower socioeconomic status (SES) areas have lower breast cancer survival but it is not clear how differences in the quality of care received contribute to these disparities. We compared adherence to clinical practice guidelines (CPG) for the diagnosis and treatment of breast cancer and subsequent breast cancer survival between women residing in lower versus higher SES areas.

**Methods:**

We conducted a multicentre population-based study of all new cases of invasive breast cancer in women diagnosed 2010–2014 in six Spanish provinces with population-based cancer registries (n=3206). Clinical data were extracted in the framework of the European Cancer High Resolution studies and vital status follow-up covered a minimum of 5 years. SES of the patient’s residence was measured with the 2011 Spanish Deprivation Index. Adherence to CPG was measured with 16 indicators based on European and Spanish guidelines. Relative survival was modelled using flexible parametric models.

**Results:**

There were no differences in the type of treatment received but women living in the lowest SES areas were less likely to undergo a sentinel lymph node biopsy, reconstruction after mastectomy, surgery within 30 days after pathological diagnosis and adjuvant treatment within 6 weeks after surgery. After accounting for demographic and clinical factors, women residing in lower SES areas had higher risk of death, HR=1.57 (95% CI 1.04, 2.36). Further accounting for adherence to CPG in the model, in particular having undergone a sentinel lymph node biopsy, eliminated the significant effect of SES.

**Conclusions:**

Despite the overall coverage of the Spanish health system, women living in more deprived areas were less likely to receive care in line with CPG and had shorter survival.

**WHAT IS ALREADY KNOWN ON THIS TOPIC**

- Findings from Europe and beyond indicate that women residing in lower socioeconomic status (SES) areas have lower breast cancer survival, suggesting that there may be disparities in the quality of care received.

**WHAT THIS STUDY ADDS**

- We investigated socioeconomic inequalities in breast cancer care based on a comprehensive assessment of clinical practice guidelines for diagnosis and treatment and using population-based data.
- Women living in more deprived areas were less likely to receive care in line with clinical practice guidelines and had shorter survival: they received similar but less timely treatments and were less likely to undergo sentinel lymph node biopsy for staging or reconstruction after mastectomy.
- Women residing in more deprived areas had higher risk of death, equivalent to 1 year of life lost.

**HOW THIS STUDY MIGHT AFFECT RESEARCH, PRACTICE OR POLICY**

- A multidisciplinary and multilevel approach is needed to ensure equitable breast cancer care, starting with the identification of the structural and individual-level factors contributing to deviations from recommended care among patients residing in lower SES areas.

## Introduction

 Breast cancer is the most common cancer worldwide, with almost 2.3 million new cases in 2022.^1^ Thanks to advances in early diagnosis and treatment, survival rates for breast cancer have considerably improved.^2^ However, there are persistent social inequalities, such that patients with lower socioeconomic status (SES) have lower survival.^3^ Such results have been found in several European countries and using diverse measures of SES. These include individual-level indicators like education, income or occupation^3^ and area-based deprivation indices that use the socioeconomic level of the person’s area of residence as a measure of SES.^4^,^9^

Area-based deprivation indices have the advantage of combining information about multiple SES indicators and are especially suited to capture health disparities arising from the socioeconomic context (eg, access to high-quality healthcare facilities).^10^ In addition, if the deprivation index is constructed for small residential areas, it is likely to reflect the individual’s SES in terms of income or education.^10^ Thus, in the context of breast cancer, using a small-area deprivation index as an indicator of patients’ SES can help capture disparities arising due to contextual and individual factors that play a role in diagnosis and treatment.

Inequalities in breast cancer survival have been attributed to diverse factors including less access to screening, later stage at diagnosis, more comorbidities and less access to treatment among women with lower SES.^11^ Earlier studies have shown that women residing in lower SES are less likely to receive traditional cancer treatments such as surgery, chemotherapy or radiotherapy and are more likely to undergo radical mastectomy instead of breast-conserving surgery.^59 12^,^14^ Some evidence suggests that women residing in lower SES areas may also be less likely to receive novel biological and precision therapies.^15^

However, previous studies that specifically evaluated to what extent care disparities contribute to socioeconomic inequalities in survival found mixed results,^11^ suggesting that socioeconomic inequalities may be strongly dependent on context.^59 12^,^14^ In addition, most previous studies focused on the receipt of specific treatment modalities, without a more comprehensive evaluation of whether the best recommended care was received.

What is considered best care is defined in clinical practice guidelines (CPGs) that aim to achieve the best results for patients and reduce unwarranted variability in diagnostic testing and treatment. Patients with breast cancer who receive care adherent to CPGs have increased overall and disease-free survival.^16^ The goal of the current research was to investigate socioeconomic inequalities in adherence to CPGs for the diagnosis and treatment of breast cancer and their implication for survival using a small-area deprivation index as a measure of SES.

## Method

We conducted an observational, analytical, multicentre, population-based study with longitudinal follow-up using data from the European Cancer High Resolution (ECHR) studies.^17 18^ We included all new cases of invasive breast cancer (C50 according to International Classification of Diseases for Oncology, 3rd Edition) in women over 15 years of age, diagnosed between 2010 and 2014 in six Spanish provinces that have population-based cancer registries: Castellón, Gipuzkoa, Girona, Granada, Navarra and Tarragona (online supplemental figure S1). The population covered represents 2 210 226 women, 9.3% of the total population of women in Spain. Each registry was required to submit a minimum of 1 year of complete incidence during the period 2010–2014, with a minimum of 500 cases and the requirement to extend the data collection period if this minimum was not reached. When information on synchronous breast tumours was submitted by the registries (ie, multiple primary tumours diagnosed within 6 months of each other), we considered the tumour with more advanced stage, or, in case of equal stage, the tumour with earlier date of diagnosis.

Cases were registered using the procedures of the International Agency for Research on Cancer, the International Association of Cancer Registries and the European Network of Cancer Registries. Additional clinical characteristics were collected using the standardised protocol of the ECHR studies.

### Demographic and clinical data

Age was categorised into four groups: 15–49, 50–69, 70–79 and 80+ years old. Comorbidity was categorised into three categories based on the 19 diseases considered in the Charlson index^19^: no comorbidities, at least one comorbidity and unknown number of comorbidities. Stage at diagnosis was derived from pathological and clinical TNM (tumour-node-metastasis) classification, 7th edition. Tumour grade of differentiation was categorised into four groups (1, 2, 3/4 and unknown). Mode of detection was recorded as symptomatic, screen detected or unknown. Tumour immunohistochemical type was categorised as oestrogen receptor/progesterone receptor positive (ER+ or PR+ but HER2−), HER2 positive (HER2+ regardless of ER/PR status), triple negative (ER, PR− and HER2−) and unclassified (due to missing data on some receptor status).

### Socioeconomic status

We used the Spanish Deprivation Index of the Spanish Society of Epidemiology.^20^ It is a small-area index based on the 2011 Spanish census and includes information from six indicators: percentage of manual workers, percentage of occasional workers, unemployment rate, percentage of the overall population with low education, percentage of young people with low education and percentage of homes without internet access.^20^ The index is assigned on the census tract level (mean area of 14.04 km^2^ (5.42 mi^2^) with average population of 1311 inhabitants) and is divided in quintiles (Q) based on its distribution across Spain, where Q1 represents the least deprived areas (highest SES) and Q5 the most deprived areas (lowest SES) (online supplemental figure S1). The addresses of patients at the moment of diagnosis were geocoded and each patient was assigned the deprivation quintile of their census tract of residence.

### Adherence to CPGs

Following a methodology used in EUROCARE,^21^ the recommendations for breast cancer diagnosis and treatment of the European^22^ and Spanish^23^ Societies of Medical Oncology, and regional Spanish guidelines regarding the timeliness of treatment,^24^ valid at the time of diagnosis of the study population, were reviewed by the multidisciplinary research team. A set of recommended care indicators was constructed, definitions were operationalised and checked against the availability of data in the ECHR studies protocol to determine whether adherence could be evaluated. 16 indicators were viable (online supplemental table S1), and for each indicator adherence was defined as receiving care in line with the recommendation (yes vs no) for those patients to whom it applied.

### Vital status follow-up

This was conducted through record linkage with the National Death Index (which includes all official death certificates in the Spanish National Institute of Statistics), record linkage with regional mortality registries and electronic healthcare records databases and through active search in the medical records of those cases considered to have a worse prognosis. Cases were followed up until 31 December 2019 in Girona, Navarra and Tarragona, and until 31 December 2021 in Castellón, Gipuzkoa and Granada. All cases had a minimum follow-up time of 5 years. Cases reported only with a death certificate (seven cases) and those identified by autopsy (one case) were excluded from the analysis.

### Statistical analysis

The distribution of demographic and clinical variables was compared across the different SES quintiles using χ^2^ tests. The effect of SES on adherence to each CPG indicator was examined unadjusted and adjusted for age and year of diagnosis by testing for a systematic increase or decrease across SES levels (p trend) and using logistic regression. ORs and rate ratios were calculated to illustrate differences between the most extreme groups (Q5 vs Q1).

Observed survival (OS) was directly derived from data using the Kaplan-Meier estimator and was calculated at 1, 3 and 5 years since diagnosis.

The marginal relative survival (MRS) was computed at 1, 3 and 5 years from diagnosis for each SES quintile, adjusting for age and year of diagnosis. The ‘standsurv’ package in Stata was used to calculate the MRS.^25^

Relative survival was modelled using flexible parametric models with restricted cubic splines.^26 27^ The specific lifetable of all-cause mortality by province, age, calendar year and SES quintile was used to correct for the expected basal hazard. This lifetable has been constructed for the Spanish population referring to the period 2011–2013.^28^ Optimal df of the cubic splines for baseline hazard function were determined by comparing the Akaike information criterion (AIC) of models with df 2–5 and retaining the model with lower value of AIC. Furthermore, a time-dependent term for age at diagnosis was included in the model using the same strategy to determinate the optimal df of the time-dependent effect. The ‘stpm2’ package of Stata software was used to construct the flexible parametric models.

A baseline survival model included SES quintile, age at diagnosis, year of diagnosis, tumour immunohistochemical type and grade of differentiation. Based on previous literature suggesting potential contributors to the effect of SES on cancer survival,^11^ the following variables were then added to the model in the following order and one at a time: comorbidities, mode of detection and stage at diagnosis. In one final step, we added those adherence indicators that (1) showed significant differences as a function of SES quintile and (2) applied to the large majority (eg, >80%) of patients. At each step, we recorded the change in the effect of SES quintile in terms of its HR and 95% CIs. Due to the lower number of patients residing in low-income areas, the Q4 and Q5 categories were grouped to improve the efficiency of the flexible parametric model.

Because many of the adherence indicators applied only to a selection of patients, we conducted sensitivity analysis to investigate the effect of adherence on survival in the baseline model further adjusted for stage and only in the population to which they applied.

Finally, years of life lost due to SES were computed as the difference in life expectancy between the least deprived (Q1) and the most deprived (Q5) groups at the mean age of diagnosis, derived from non-linear predictions in the baseline flexible parametric model.^29^

### Patient and public involvement

Patients or the public were not involved in the design, or conduct, or reporting or dissemination plans of this research.

## Results

The study included 3206 patients with breast cancer, with a mean age at diagnosis of 60.6 years. The majority of women (72.5%) were diagnosed at stages I and II and 28.6% had died at the end of the follow-up period. The majority of women residing in high SES areas were from the provinces of Gipuzkoa, Navarra and Girona, whereas the majority of women residing in the lowest SES areas were from Granada. Women residing in more deprived areas had more comorbidities, were less likely to have been diagnosed through screening and had later stages at diagnosis (table 1). There were larger proportions of unknown or unclassified immunohistochemical results among women residing in higher SES areas due to one relatively high SES province not having collected data on immunohistochemistry (Gipuzkoa). The majority of women had ER+/ER− (62.2%) or HER+ (13.4%) tumours, with a minority diagnosed with the more aggressive triple negative tumours (7.4%).

**Table 1:** Distribution (number and percentage) of patients with breast cancer as a function of demographic and clinical characteristics in each socioeconomic status (SES) quintile (n=3206)

			SES quintile					
		Total (%)	Q1 (highest SES) (%)	Q2 (%)	Q3 (%)	Q4 (%)	Q5 (lowest SES) (%)	P value
Overall		3206 (100)	665 (100.0)	912 (100.0)	813 (100.0)	510 (100.0)	306 (100.0)	**<0.001**
Province	Castellón	559 (17.4)	57 (8.6)	109 (12.0)	179 (22.0)	162 (31.8)	52 (17.0)	**<0.001**
	Gipuzkoa	453 (14.1)	167 (25.1)	177 (19.4)	91 (11.2)	18 (3.5)	0 (0.0)	
	Girona	761 (23.7)	128 (19.2)	272 (29.8)	206 (25.3)	108 (21.2)	47 (15.4)	
	Granada	450 (14.0)	81 (12.2)	83 (9.1)	57 (7.0)	74 (14.5)	155 (50.7)	
	Navarra	441 (13.8)	139 (20.9)	121 (13.3)	105 (12.9)	59 (11.6)	17 (5.6)	
	Tarragona	542 (16.9)	93 (14.0)	150 (16.4)	175 (21.5)	89 (17.5)	35 (11.4)	
Age group	15–49	899 (28.0)	199 (29.9)	260 (28.5)	209 (25.7)	158 (31.0)	73 (23.9)	**0.009**
	50–69	1419 (44.3)	314 (47.2)	405 (44.4)	346 (42.6)	218 (42.7)	136 (44.4)	
	70–79	490 (15.3)	84 (12.6)	121 (13.3)	151 (18.6)	77 (15.1)	57 (18.6)	
	80+	398 (12.4)	68 (10.2)	126 (13.8)	107 (13.2)	57 (11.2)	40 (13.1)	
Year	2010	547 (17.1)	93 (14.0)	182 (20.0)	168 (20.7)	69 (13.5)	35 (11.4)	**<0.001**
	2011	1908 (59.5)	388 (58.3)	548 (60.1)	446 (54.9)	298 (58.4)	228 (74.5)	
	2012	310 (9.7)	45 (6.8)	61 (6.7)	94 (11.6)	84 (16.5)	26 (8.5)	
	2013	376 (11.7)	121 (18.2)	105 (11.5)	86 (10.6)	51 (10.0)	13 (4.2)	
	2014	65 (2.0)	18 (2.7)	16 (1.8)	19 (2.3)	8 (1.6)	4 (1.3)	
Comorbidity	0	1045 (32.6)	307 (46.2)	326 (35.7)	225 (27.7)	120 (23.5)	67 (21.9)	**<0.001**
	≥1	1216 (37.9)	232 (34.9)	341 (37.4)	300 (36.9)	178 (34.9)	165 (53.9)	
	Unknown	945 (29.5)	126 (18.9)	245 (26.9)	288 (35.4)	212 (41.6)	74 (24.2)	
Mode of detection	Symptomatic	1928 (60.1)	397 (59.7)	527 (57.8)	489 (60.1)	313 (61.4)	202 (66.0)	**0.003**
	Screened	1207 (37.6)	242 (36.4)	361 (39.6)	310 (38.1)	191 (37.5)	103 (33.7)	
	Unknown	71 (2.2)	26 (3.9)	24 (2.6)	14 (1.7)	6 (1.2)	1 (0.3)	
Immunohistochemical profile	ER/PR+	1994 (62.2)	349 (52.5)	531 (58.2)	551 (67.8)	361 (70.8)	202 (66.0)	**<0.001**
	HER2+	430 (13.4)	103 (15.5)	123 (13.5)	96 (11.8)	68 (13.3)	40 (13.1)	
	Triple negative	237 (7.4)	45 (6.8)	61 (6.7)	60 (7.4)	36 (7.1)	35 (11.4)	
	Unclassified	545 (17.0)	168 (25.3)	197 (21.6)	106 (13.0)	45 (8.8)	29 (9.5)	
Differentiation grade	1	657 (20.5)	131 (19.7)	165 (18.1)	178 (21.9)	108 (21.2)	75 (24.5)	**0.045**
	2	1306 (40.7)	267 (40.2)	394 (43.2)	338 (41.6)	212 (41.6)	95 (31.0)	
	3/4	722 (22.5)	156 (23.5)	203 (22.3)	179 (22.0)	112 (22.0)	72 (23.5)	
	Unknown	521 (16.3)	111 (16.7)	150 (16.4)	118 (14.5)	78 (15.3)	64 (20.9)	
Stage at diagnosis	I	1239 (38.6)	287 (43.2)	369 (40.5)	310 (38.1)	169 (33.1)	104 (34.0)	**<0.001**
	II	1086 (33.9)	197 (29.6)	301 (33.0)	288 (35.4)	196 (38.4)	104 (34.0)	
	III	517 (16.1)	112 (16.8)	132 (14.5)	134 (16.5)	80 (15.7)	59 (19.3)	
	IV	187 (5.8)	37 (5.6)	52 (5.7)	31 (3.8)	38 (7.5)	29 (9.5)	
	Unknown	177 (5.5)	32 (4.8)	58 (6.4)	50 (6.2)	27 (5.3)	10 (3.3)	
Surgery	Yes	2871 (89.6)	602 (90.5)	826 (90.6)	722 (88.8)	455 (89.2)	266 (86.9)	0.181
	No	316 (9.9)	58 (8.7)	81 (8.9)	83 (10.2)	54 (10.6)	40 (13.1)	
	Unknown	19 (0.6)	5 (0.8)	5 (0.5)	8 (1.0)	1 (0.2)	0 (0.0)	
Type of surgery	Not done	316 (9.9)	58 (8.7)	81 (8.9)	83 (10.2)	54 (10.6)	40 (13.1)	0.259
	BCS	1972 (61.5)	432 (65.0)	557 (61.1)	500 (61.5)	298 (58.4)	185 (60.5)	
	Mastectomy	885 (27.6)	166 (25.0)	266 (29.2)	217 (26.7)	155 (30.4)	81 (26.5)	
	Done, but unknown type of surgery	11 (0.3)	4 (0.6)	2 (0.2)	3 (0.4)	2 (0.4)	0 (0.0)	
	BCS+ mastectomy	3 (0.1)	0 (0.0)	1 (0.1)	2 (0.2)	0 (0.0)	0 (0.0)	
	Unknown	19 (0.6)	5 (0.8)	5 (0.5)	8 (1.0)	1 (0.2)	0 (0.0)	
Chemotherapy	Yes	1739 (54.2)	387 (58.2)	463 (50.8)	423 (52.0)	286 (56.1)	180 (58.8)	**0.043**
	No	1420 (44.3)	269 (40.5)	434 (47.6)	376 (46.2)	216 (42.4)	125 (40.8)	
	Unknown	47 (1.5)	9 (1.4)	15 (1.6)	14 (1.7)	8 (1.6)	1 (0.3)	
Radiotherapy	Yes	2331 (72.7)	507 (76.2)	638 (70.0)	602 (74.0)	367 (72.0)	217 (70.9)	0.136
	No	826 (25.8)	146 (22.0)	259 (28.4)	198 (24.4)	136 (26.7)	87 (28.4)	
	Unknown	49 (1.5)	12 (1.8)	15 (1.6)	13 (1.6)	7 (1.4)	2 (0.7)	
Endocrine treatment	Yes	2568 (80.1)	538 (80.9)	728 (79.8)	653 (80.3)	418 (82.0)	231 (75.5)	0.543
	No	581 (18.1)	114 (17.1)	166 (18.2)	149 (18.3)	83 (16.3)	69 (22.5)	
	Unknown	57 (1.8)	13 (2.0)	18 (2.0)	11 (1.4)	9 (1.8)	6 (2.0)	
Targeted treatment	Yes	372 (11.6)	89 (13.4)	105 (11.5)	81 (10.0)	63 (12.4)	34 (11.1)	0.388
	No	2781 (86.7)	561 (84.4)	794 (87.1)	718 (88.3)	438 (85.9)	270 (88.2)	
	Unknown	53 (1.7)	15 (2.3)	13 (1.4)	14 (1.7)	9 (1.8)	2 (0.7)	
ER positivity	Positive	1949 (60.8)	353 (53.1)	540 (59.2)	546 (67.2)	355 (69.6)	155 (50.7)	**<0.001**
	Weakly positive	300 (9.4)	56 (8.4)	57 (6.2)	53 (6.5)	58 (11.4)	76 (24.8)	
	Negative	431 (13.4)	81 (12.2)	120 (13.2)	109 (13.4)	67 (13.1)	54 (17.6)	
	Unknown	526 (16.4)	175 (26.3)	195 (21.4)	105 (12.9)	30 (5.9)	21 (6.9)	
PR positivity	Positive	1711 (53.4)	309 (46.5)	463 (50.8)	472 (58.1)	325 (63.7)	142 (46.4)	**<0.001**
	Weakly positive	302 (9.4)	55 (8.3)	70 (7.7)	57 (7.0)	56 (11.0)	64 (20.9)	
	Negative	661 (20.6)	126 (18.9)	178 (19.5)	180 (22.1)	97 (19.0)	80 (26.1)	
	Unknown	532 (16.6)	175 (26.3)	201 (22.0)	104 (12.8)	32 (6.3)	20 (6.5)	
HER2+	Not done	64 (2.0)	9 (1.4)	14 (1.5)	18 (2.2)	15 (2.9)	8 (2.6)	0.230
	0 (negative)	1294 (40.4)	261 (39.2)	354 (38.8)	344 (42.3)	203 (39.8)	132 (43.1)	
	1+ (negative)	980 (30.6)	206 (31.0)	290 (31.8)	250 (30.8)	151 (29.6)	83 (27.1)	
	2++	438 (13.7)	86 (12.9)	134 (14.7)	104 (12.8)	76 (14.9)	38 (12.4)	
	3+++ (positive)	364 (11.4)	89 (13.4)	101 (11.1)	84 (10.3)	58 (11.4)	32 (10.5)	
	Unknown	66 (2.1)	14 (2.1)	19 (2.1)	13 (1.6)	7 (1.4)	13 (4.2)	
Ki67 positivity	High	721 (22.5)	155 (23.3)	180 (19.7)	171 (21.0)	146 (28.6)	69 (22.5)	**<0.001**
	Low	793 (24.7)	124 (18.6)	169 (18.5)	209 (25.7)	161 (31.6)	130 (42.5)	
	Unknown	1692 (52.8)	386 (58.0)	563 (61.7)	433 (53.3)	203 (39.8)	107 (35.0)	

The values in bold indicate that the p-value is less than 0.05.

BCS, breast-conserving surgery; ER, oestrogen receptor; PR, progesterone receptor.

Adherence on the different indicators varied from 29.2% (Ind16) to 94.0% (Ind2) and was generally higher among younger patients, patients without comorbidities and diagnosed through screening (online supplemental table S2). Adherence also varied as a function of grade of differentiation and stage at diagnosis but without a clear pattern (online supplemental table S2).

### Socioeconomic inequalities in adherence to CPGs

Tables2  3 show that women living in the lowest SES areas were less likely to receive a sentinel lymph node biopsy (SLNB) (Ind3: p_trend_<0.001). This was also the case when we considered only women who had clinically negative axillary lymph nodes (Ind4: p_trend_=0.007). This pattern of lower rates of SLNB among women with lower SES was also present among women with clinically positive nodes (24.6% vs 30.0% for Q5 vs Q1, respectively) and with different stages at diagnosis (45.8% vs 51.0% for Q5 vs Q1 among early stages I–II and 13.6% vs 16.0% for Q5 vs Q1 among late stages III–IV).

**Table 2:** Adherence (in percentage) to each clinical practice guideline indicator overall and by socioeconomic status (SES) quintile, where Q1 reflects the highest and Q5 the lowest SES group, respectively

			SES quintile				
**Indicator definition**	n*	**Overall**	**Q1**	**Q2**	**Q3**	**Q4**	**Q5**
Ind1—the patient’s case was evaluated by the tumour-specific commission before starting treatment.	2647	84.2	84.9	83.6	82.5	84.5	88.2
Ind2—the patient underwent full tumour classification (HER, ER and PR status).	2753	94.0	94.0	93.6	94.7	94.9	92.2
Ind3—the patient underwent a sentinel lymph node biopsy (SLNB).	3206	61.3	67.7	62.2	59.5	57.3	56.5
Ind4—for patients with clinically negative axillary lymph nodes: the patient underwent SLNB.	1320	75.8	81.1	77.6	71.5	72.5	72.7
Ind5—for patients with a positive result in SLNB: the patient underwent axillary lymph node dissection (ALND).	662	77.3	70.4	74.4	82.6	80.2	83.6
Ind6—for patients undergoing ALND: the patient had 10 or more lymph nodes removed and examined.	1444	61.4	65.7	67.9	59.1	52.9	59.4
Ind7—the patient was surgically treated with breast-conserving surgery.	3206	61.5	65.0	61.1	61.5	58.4	60.5
Ind8—for patients with stage I/IIA disease: the patient was treated with conservative surgery and receiving adjuvant radiotherapy.	1923	78.0	82.3	76.8	77.5	74.4	79.3
Ind9—for patients undergoing mastectomy: the patient underwent reconstruction.	888	31.8	40.4	33.7	24.7	31.0	28.4
Ind10—for patients with HER2+: the patient received targeted treatment.	430	80.9	80.6	82.1	81.2	82.4	75.0
Ind11—for patients with HER2+: the patient received chemotherapy.	430	82.1	81.6	82.1	80.2	86.8	80.0
Ind12—for patients with ER+ or PR+: the patient received hormonal treatment.	2308	93.4	94.1	93.1	92.4	94.8	92.7
Ind13—for patients with triple negative cancer: the patient received chemotherapy.	237	80.6	86.7	77.0	83.3	77.8	77.1
Ind14 —for patients with node-positive results: the patient received chemotherapy.	1439	75.4	78.8	73.3	74.0	73.9	79.6
Ind15—for patients who underwent surgery: the patient underwent surgical intervention within 30 days after pathological diagnosis.	2871	36.1	40.0	34.6	39.6	33.6	26.3
Ind16—for patients who underwent surgery: the patient started adjuvant treatment within 6 weeks from the date of surgery.	2871	29.2	35.4	27.7	28.7	27.0	25.2

* n=number of patients to whom the indicator applied (eg, based on clinical characteristics). Two provinces did not collect the data necessary to evaluate some indicators (Castellon on evaluation of the tumour-specific commission and Gipuzkoa on immunohistochemical classification) and have not been included in the analyses of the respective indicators where these data were necessary.

ER, oestrogen receptor; PR, progesterone receptor.;

**Table 3:** Differences in adherence to each clinical practice guideline indicator as a function of socioeconomic status (SES) group, where Q1 reflects the highest and Q5 the lowest SES group

	Unadjusted			Adjusted by age and year of diagnosis		
Indicator definition	P trend	OR Q5 vs Q1 (95% CI)	RR Q5 vs Q1 (95% CI)	P trend	OR Q5 vs Q1 (95% CI)	RR Q5 vs Q1 (95% CI)
Ind1—the patient’s case was evaluated by the tumour-specific commission before starting treatment.	0.191	1.21 (0.66 to 2.35)	1.01 (0.97 to 1.05)	**0.037**	1.49 (0.79 to 2.96)	1.04 (0.88 to 1.21)
Ind2—the patient underwent full tumour classification (HER, ER and PR status).	0.829	0.75 (0.43 to 1.32)	0.98 (0.94 to 1.02)	0.916	0.81 (0.46 to 1.43)	0.96 (0.79 to 1.17)
Ind3—the patient underwent a sentinel lymph node biopsy (SLNB).	**<0.001**	**0.60 (0.45 to 0.79)**	**0.83 (0.74 to 0.93)**	**<0.001**	**0.64 (0.48 to 0.87)**	0.87 (0.72 to 1.03)
Ind4—for patients with clinically negative axillary lymph nodes: the patient underwent SLNB.	**0.007**	**0.61 (0.39 to 0.98)**	**0.90 (0.80 to 1.00)**	**0.010**	0.61 (0.36 to 1.05)	0.93 (0.74 to 1.16)
Ind5—for patients with a positive result in SLNB: the patient underwent axillary lymph node dissection (ALND).	**0.008**	**2.09 (1.04 to 4.53)**	**1.18 (1.02 to 1.36)**	**0.023**	1.90 (0.92 to 4.21)	1.16 (0.83 to 1.60)
Ind6—for patients undergoing ALND: the patient had 10 or more lymph nodes removed and examined.	0.989	0.88 (0.53 to 1.49)	0.97 (0.86 to 1.10)	0.928	0.92 (0.55 to 1.56)	1.00 (0.77 to 1.29)
Ind7—the patient was surgically treated with breast-conserving surgery.	0.060	0.82 (0.62 to 1.09)	0.93 (0.84 to 1.04)	0.133	0.88 (0.65 to 1.18)	0.95 (0.79 to 1.13)
Ind8—for patients with stage I/IIA disease: the patient was treated with conservative surgery and receiving adjuvant radiotherapy.	0.127	0.83 (0.53 to 1.29)	0.96 (0.88 to 1.05)	0.108	0.81 (0.52 to 1.30)	0.96 (0.79 to 1.17)
Ind9—for patients undergoing mastectomy: the patient underwent reconstruction.	**0.024**	0.59 (0.33 to 1.05)	0.71 (0.48 to 1.05)	0.167	0.82 (0.41 to 1.64)	0.87 (0.53 to 1.38)
Ind10—for patients with HER2+: the patient received targeted treatment.	0.430	0.61 (0.25 to 1.53)	0.90 (0.74 to 1.10)	0.280	0.51 (0.18 to 1.46)	0.90 (0.58 to 1.37)
Ind11—for patients with HER2+: the patient received chemotherapy.	0.813	0.86 (0.35 to 2.27)	0.97 (0.81 to 1.16)	0.953	0.65 (0.20 to 2.23)	0.94 (0.61 to 1.42)
Ind12—for patients with ER+ or PR+: the patient received hormonal treatment.	0.803	0.74 (0.37 to 1.54)	0.98 (0.95 to 1.02)	0.752	0.73 (0.36 to 1.53)	0.98 (0.83 to 1.16)
Ind13—for patients with triple negative cancer: the patient received chemotherapy.	0.400	0.52 (0.15 to 1.66)	0.89 (0.72 to 1.10)	0.690	0.59 (0.14 to 2.37)	0.93 (0.55 to 1.58)
Ind14—for patients with node-positive results: the patient received chemotherapy.	0.901	1.05 (0.65 to 1.74)	1.01 (0.91 to 1.12)	0.324	1.56 (0.83 to 3.00)	1.05 (0.84 to 1.31)
Ind15—for patients who underwent surgery: the patient underwent surgical intervention within 30 days after pathological diagnosis.	**0.002**	**0.53 (0.39 to 0.73)**	**0.66 (0.53 to 0.82)**	**<0.001**	**0.51 (0.37 to 0.70)**	**0.65 (0.49 to 0.84)**
Ind16—for patients who underwent surgery: the patient started adjuvant treatment within 6 weeks from the date of surgery.	**0.002**	**0.64 (0.46 to 0.89)**	**0.75 (0.60 to 0.94)**	**0.009**	**0.71 (0.50 to 1.00)**	0.79 (0.59 to 1.04)

P-trend values test for a systematic change across SES levels considering three classification groups: adherence, non-adherence and missing. ORs are from logistic regressions that consider two classification groups: adherence versus non-adherence, excluding patients with missing data. The values in bold indicate that the associated p-value is less than 0.05.

ER, oestrogen receptor; PR, progesterone receptor; RR, risk ratio.;

In contrast, among patients with a positive result in sentinel lymph nodes, women residing in the lowest SES areas were more likely to undergo axillary lymph node dissection (Ind5: p_trend_=0.008). Patients from the lowest SES areas were also less likely to undergo reconstruction after mastectomy (Ind9: p_trend_=0.024), although this trend disappeared after adjustment for age and year of diagnosis.

Finally, women living in the lowest SES areas were less likely to receive timely treatment, such as undergoing surgery within 30 days after pathological diagnosis (Ind15: p_trend_=0.002) or starting adjuvant treatment within 6 weeks after surgery (Ind16: p_trend_=0.002). In the case of Ind15, having received neoadjuvant treatment was associated with less adherence to Ind15 (p<0.001; ie, time to surgery was longer). However, the effect of SES remained significant and in the same direction after considering reception of neoadjuvant treatment (p=0.002).

In contrast, there was little difference between the SES groups in the reception of tumour classification tests (Ind2), the number of lymph nodes removed and examined during axillary lymph node dissection (Ind6), breast-conserving surgery (Ind7) and the recommended adjuvant treatments for the different tumour types (Ind8–13).

### Socioeconomic inequalities in survival

At 1 year, OS was 96.8% for women living in the highest SES areas and 93.5% for women living in the lowest SES areas. The gap widened overtime, with 5-year OS=85.6% for women residing in the least deprived areas versus OS=78.4% for women residing in the most deprived areas (online supplemental table S3).

MRS, adjusted for age and year of diagnosis, showed a similar pattern. At 1 year, MRS was 97.8% (95% CI 96.9% to 98.7%) for women living in the highest SES areas and 96.4% (95% CI 94.9% to 98.0%) for women living in the lowest SES areas. The gap widened overtime, with 5-year MRS=92.4% (95% CI 89.9% to 95.0%) for women residing in the least deprived areas versus MRS=88.0% (95% CI 83.7% to 92.5%) for women residing in the most deprived areas (online supplemental table S4).

In the baseline relative survival model, adjusted for age, year of diagnosis, immunohistochemical type and tumour grade, women residing in the lowest SES areas (Q4–Q5) had a higher risk of death (HR=1.54, 95% CI 1.02 to 2.35) than women residing in the highest SES areas (model 1 in table 4). Derived from this model, at mean age of diagnosis (60.6 years old) and after accounting for tumour characteristics, low SES status was associated with 0.98 years of life lost, with the remaining life expectancy being 34.6 years for women living in the highest SES areas (Q1) and 33.6 years for women living in the lowest SES areas (Q5). The higher risk of death among women residing in lowest SES areas (Q4 or Q5) remained relatively unchanged after further adjusting for comorbidity, mode of detection and stage at diagnosis, with HR=1.57 (95% CI 1.04 to 2.36) for Q5–Q4 vs Q1 (model 4 in table 4).

**Table 4:** Results from flexible parametric models of relative survival

		Model 1	Model 2	Model 3	Model 4	Model 5A	Model 5B	Model 5C
Socioeconomic status quintile	Q2 vs Q1	1.17 (0.77 to 1.78)	1.16 (0.76 to 1.77)	1.31 (0.86 to 1.98)	1.41 (0.94 to 2.11)	1.23 (0.83 to 1.84)	1.41 (0.94 to 2.10)	1.46 (0.97 to 2.19)
	Q3 vs Q1	0.69 (0.41 to 1.15)	0.68 (0.41 to 1.13)	0.74 (0.45 to 1.22)	0.83 (0.51 to 1.35)	0.80 (0.50 to 1.28)	0.90 (0.57 to 1.43)	0.99 (0.62 to1.58)
	Q4–Q5 vs Q1	**1.54 (1.02 to 2.35)**	1.50 (0.99 to 2.29)	**1.55 (1.02 to 2.36)**	**1.57 (1.04 to 2.36)**	1.43 (0.95 to 2.14)	**1.52 (1.02 to 2.26)**	**1.57 (1.05 to 2.37)**
Age at diagnosis (1-year increment)		**1.01 (1.00 to 1.03)**	**1.01 (1.00 to 1.02)**	**1.01 (1.00 to 1.02)**	**1.01 (1.00 to 1.02)**	1.01 (1.00 to 1.02)	1.00 (0.99 to 1.01)	0.99 (0.98 to 1.00)
Year of diagnosis (1-year increment)		0.88 (0.73 to 1.06)	0.94 (0.78 to 1.14)	0.96 (0.80 to 1.16)	1.05 (0.88 to 1.25)	1.03 (0.86 to 1.22)	1.01 (0.86 to 1.20)	1.02 (0.86 to 1.20)
Immunohistochemical type	Triple negative versus ER/PR+	**2.64 (1.77 to 3.93)**	**2.60 (1.74 to 3.87)**	**2.49 (1.69 to 3.67)**	**2.50 (1.71 to 3.66)**	1.40 (0.95 to 2.07)	1.19 (0.80 to 1.78)	1.20 (0.80 to 1.80)
	HER2+ versus ER/PR+	1.23 (0.79 to 1.91)	1.25 (0.80 to 1.94)	1.12 (0.72 to 1.74)	0.94 (0.61 to 1.43)	**1.55 (1.03 to 2.33)**	1.51 (1.00 to 2.28)	1.48 (0.98 to 2.24)
	Unclassified versus ER/PR+	**1.89 (1.27 to 2.81)**	**2.10 (1.40 to 3.15)**	**1.84 (1.23 to 2.75)**	**1.82 (1.23 to 2.70)**	**3.83 (1.53 to 9.62)**	**2.61 (1.09 to 6.20)**	2.32 (0.98 to 5.50)
Grade of differentiation	2 vs 1	4.60 (0.89 to 23.75)	4.09 (0.95 to 17.69)	2.79 (0.95 to 8.20)	1.17 (0.55 to 2.53)	**2.20 (1.31 to 3.67)**	**2.06 (1.22 to 3.48)**	**2.02 (1.18 to 3.45)**
	3/4 vs 1	**15.26 (2.98 to 78.17)**	**13.62 (3.18 to 58.34)**	**8.29 (2.86 to 24.00)**	**3.08 (1.46 to 6.50)**	**2.34 (1.60 to 3.42)**	**3.88 (2.57 to 5.86)**	**3.34 (2.21 to 5.06)**
	Unknown versus 1	**21.13 (4.16 to 107.38)**	**18.91 (4.45 to 80.39)**	**10.50 (3.65 to 30.25)**	**3.39 (1.60 to 7.17)**	0.88 (0.58 to 1.34)	1.14 (0.75 to 1.75)	1.13 (0.74 to 1.72)
Comorbidity	1 or more versus none		1.27 (0.85 to 1.92)	1.36 (0.91 to 2.03)	1.45 (0.98 to 2.14)	**1.60 (1.08 to 2.37)**	1.41 (0.95 to 2.09)	1.41 (0.95 to 2.10)
	Unknown versus none		**1.62 (1.06 to 2.46)**	**1.56 (1.03 to 2.35)**	**1.78 (1.19 to 2.66)**	1.41 (0.65 to 3.09)	1.11 (0.53 to 2.32)	1.20 (0.59 to 2.47)
Mode of detection	Symptomatic versus screen detected			**5.93 (2.72 to 12.91)**	**3.39 (1.43 to 8.00)**	**3.24 (1.50 to 6.99)**	**2.47 (1.19 to 5.11)**	**2.58 (1.27 to 5.25)**
	Unknown versus screen detected			**3.69 (2.24 to 6.07)**	**2.43 (1.45 to 4.09)**	**3.38 (1.57 to 7.29)**	1.84 (0.91 to 3.76)	1.95 (0.97 to 3.93)
Stage at diagnosis	II vs I				6.50 (0.73 to 58.24)	2.16 (0.59 to 7.95)	11.18 (0.29 to 426.12)	8.90 (0.75 to 105.31)
	III/IV vs I				**57.86 (6.65 to 503.31)**	**12.26 (3.48 to 43.24)**	**60.69 (1.61 to 2293.83)**	**46.78 (4.04 to 542.34)**
	Unknown versus I				**27.09 (2.93 to 250.19)**	**6.51 (1.71 to 24.72)**	23.76 (0.61 to 923.62)	**18.10 (1.50 to 217.93)**
Ind3*	Adherence versus non-adherence					**0.21 (0.12 to 0.38)**		
	Unknown versus non-adherence					0.65 (0.27 to 1.60)		
Ind15†	Adherence versus non-adherence						0.81 (0.49 to 1.36)	
	Unknown versus non-adherence						**7.22 (5.03 to 10.36)**	
	Not applicable versus non-adherence						**8.57 (2.27 to 32.41)**	
Ind16‡	Adherence versus non-adherence							0.69 (0.38 to 1.26)
	Unknown versus non-adherence							**10.25 (6.72 to 15.64)**
	Not applicable versus non-adherence							**3.75 (2.39 to 5.88)**

Mortality HR and 95% CI (n=3206). The values in bold indicate that the associated p-value is less than 0.05.

* The patient underwent sentinel lymph node biopsy.

† For patients in whom surgery is indicated: the patient underwent surgical intervention within 30 days after pathological diagnosis.

‡ For patients who underwent surgery: the patient started adjuvant treatment within 6 weeks from the date of surgery.

ER, oestrogen receptor; PR, progesterone receptor.

To examine the role of adherence to CPG, we then added to the model the three adherence indicators which applied to the majority of patients and showed differences as a function of SES: Ind3, Ind15 and Ind16 (models 5A–5C in table 4). The effect of SES was largely maintained after including the timeliness of cancer indicators (Ind15 and Ind16, models 5B and 5C, respectively) but disappeared after including Ind3 (model 5A). Having undergone an SLNB (Ind3) was associated with lower risk of death, HR=0.21 (95% CI 0.12 to 0.38) (model 5A), whereas the timeliness indicators had no significant effects (models 5B and 5C). The results remained similar when we added the three indicators at once to the same model. In the fully adjusted models 5A–5C, the risk of death was higher among patients who had had an HER2+ or unclassified immunohistochemical profile (vs ER/PR+), poorly differentiated tumours, comorbidities, a tumour that was not screen detected and an advanced or unknown stage at diagnosis.

The results from the sensitivity analysis of the effect of the separate indicators on survival are reported in online supplemental table S5. Adherence was generally consistently associated with lower risk of mortality, significantly so for 8 out of the 16 indicators.

## Discussion

This study investigated equity in breast cancer care based on a comprehensive assessment of CPGs and in relation to socioeconomic deprivation. Despite the overall coverage of the Spanish healthcare system, women living in more deprived areas were less likely to receive care in line with CPGs and had shorter survival. This corresponded to 1 year of life lost among those with low SES, after taking into account age and tumour characteristics.

We did not find major differences in the type of treatment received as a function of SES. Instead, we found that women living in more deprived areas were less likely to undergo an SLNB and reconstruction after mastectomy and experienced longer delays in receiving surgery and adjuvant treatment. In a model adjusted for multiple factors, having undergone an SLNB was associated with better survival. In contrast, the indicators assessing the timeliness of treatment had no significant effects on survival. Overall, our analysis shows an important socioeconomic ingredient in both adherence to CPG and survival.

Two previous studies—one from Europe^30^ and one from the USA^31^—both on women diagnosed prior to 2010, reported that lower SES was associated with a lower likelihood of receiving an SLNB. SLNB has become a standard of care for patients with breast cancer due to its minimally invasive nature and ability to accurately stage the axilla. It causes significantly less morbidity compared with traditional axillary dissection and has been hypothesised to contribute to improved survival through more accurate staging and better axillary tumour control.^32^ Studies examining this procedure in the context of survival are relatively rare,^33^ with the current study adding novel evidence in this respect. Women with lower SES were less likely to undergo SLNB, but they were more likely to undergo axillary lymph node dissection after a positive SLNB. The latter could be due to more advanced or aggressive disease in this group, among others.

At the time of the diagnosis of the study patients, SLNB had already been part of CPGs for several years. However, the facilities and expertise necessary for the procedure were not yet available in many regional hospitals in Spain. It is possible that patients treated in hospitals in more remote and/or deprived areas had to be referred to a larger treatment centre to have the procedure. This may have decreased the rate of SLNB for diverse reasons (eg, medical professionals foregoing it to reduce delays, patients opting out due to diverse barriers), thereby giving rise to the observed socioeconomic inequalities. Recent research from Spain confirms the importance of the resources available at the treating centre, showing that patients with breast cancer treated in smaller hospitals have lower survival.^34^ This interpretation would be also in line with the ‘inverse equity hypothesis’, stating that new healthcare interventions initially reach those of higher SES and only later people from more disadvantaged groups.^35^ According to this hypothesis, the introduction of new CPGs would result in an early increase in inequity ratio for coverage and other health outcomes that would be reduced only when the more disadvantaged groups eventually gain access. According to this, we would expect later evidence to show that the observed inequalities in SLNB have diminished or disappeared, now that the procedure has become widely implemented in more hospitals across Spain.

Previous studies investigating if women with low SES have longer waiting times to first-line therapy found little differences.^8 36 37^ We found that women living in more deprived areas were less likely to undergo surgery within 30 days after diagnosis, regardless of neoadjuvant treatment, and were less likely to start adjuvant treatment within 6 weeks after surgery. However, adherence to these timeliness of care indicators was unrelated to survival in the fully adjusted model. Previous research shows that delaying surgery more than 12 weeks is associated with worse survival^38^; however, the interval targeted by the CPGs was much shorter. Previous studies also show that delaying adjuvant treatment for more than 4 weeks can reduce survival, but only for patients with triple negative tumours, which constitute a small proportion of the current sample.^39^

Women residing in lower SES areas were less likely to undergo reconstruction after mastectomy. This is in line with findings from other European countries showing that, despite universal access to healthcare, low SES is related to lower rates of overall or immediate reconstruction.^40 41^ Reconstruction was less frequently performed on older patients (online supplemental table S2), and the proportion of older patients undergoing mastectomy (>80 years old) was higher in the lowest (21%) versus highest (8.4%) SES areas. Thus, the higher number of young patients residing in high SES areas is most likely contributing to the observed SES differences that became non-significant after adjusting for age. More advanced disease and/or radiotherapy among women with lower SES could be contributing as well.^40^ Women with lower SES might also be more likely to opt out of reconstruction due to financial concerns (eg, longer time off work needed) or be less likely to receive information regarding reconstruction options.^40^ Last but not least, breast reconstruction would be offered mostly in large reference care centres with the necessary expertise and plastic surgery facilities and would require additional healthcare visits and/or further surgeries. This could generate additional travel or financial barriers (eg, due to missing work) for women from lower SES areas.

Sensitivity analysis investigating the effects of adherence to the different indicators on survival detected significant beneficial effects on eight out of the 16 indicators. For the majority of indicators without significant effects, either the sample size was small (Ind5, Ind10 and Ind11) or adherence was very high (Ind1 and Ind2), making it less likely to detect significant effects. Overall, the general pattern of results is consistent with previous work documenting positive effects of adherence to CPGs.^16^

The current study adds novel results for Spain based on population-based data, showing that women from lower SES areas have lower observed and relative breast cancer survival. Even after the introduction and generalisation of new breast cancer therapies in the Spanish healthcare system, traditional factors such as stage at diagnosis^42^ and easier access to healthcare facilities^43^ continue to influence survival. Net survival at 5 years in the current study was higher (92.3%) than that estimated by the Spanish Network of Cancer Registries^44^ (92.3%), which could be due to differences in methodology or lower representation of regions with higher deprivation in the current study.

The current research provided an in-depth examination of diagnostic and staging procedures and the reception of personalised treatment. Major strengths of this study included its population-based nature and the use of lifetables based on SES to estimate relative survival. It was nevertheless limited by missing data in some of the participating provinces that reduced the sample size for some indicators or prevented more accurate molecular classification. We adjusted for multiple clinical factors; however, we had no information about other relevant variables that could influence the socioeconomic gradient in survival such as health-related lifestyle,^45^ adherence to hormonal treatment,^46^ rurality of the patient’s residence or its distance from the healthcare facility. The examination of adherence to CPGs was confined only to indicators that could be evaluated with the available data. We had no information regarding the reasons for not undergoing certain procedures or treatments, which may have been due to professional recommendation and/or patient preferences, among others.

The study was based on patients diagnosed 10 or more years ago, which could reduce its relevance to current clinical practice. Many of the indicators would still be relevant to evaluate CPGs; however, others have evolved to more personalised treatments. To illustrate, there has been a de-escalation process in the use of axillary lymph node dissection after a positive SLNB (Ind5), such that it may now be recommended only for a subset of patients.^47^ Depending on the tumour type and extension, additional treatments may be recommended such as kinase inhibitors or neoadjuvant immunotherapy (Ind12 and Ind13), or chemotherapy may be omitted depending on risk classification results (Ind14).^47^ The study nevertheless contributes more recent and comprehensive clinical data compared with most previous population-based studies on socioeconomic inequalities in care and survival in Europe that were based on patients diagnosed before 2010.

Research shows that both contextual and individual factors contribute to socioeconomic inequalities in cancer outcomes.^48^ The use of a deprivation index as a proxy for individual SES inevitably introduces measurement error because the deprivation level of the person’s area of residence may not accurately capture the person’s access to healthcare resources and would not always coincide with their individual-level SES.^48^ Whereas deprivation indices are considered good at capturing inequalities stemming from contextual factors, they may obscure or fail to detect differences arising due to individual-level factors (eg, the ability to effectively use healthcare, diverse experiences with healthcare providers, etc).^10^

Understanding and reducing socioeconomic inequalities is a priority in Europe’s Beating Cancer Plan.^49^ This study has implications for the reduction of disparities in clinical practice and breast cancer survival. The reasons behind the documented inequalities are likely multifactorial.^50^ For instance, patients residing in lower SES areas may be treated in hospitals in which some of the recommended procedures or treatments are not (yet) available due to lack of technical or human resources. Many of the lower SES areas may also be less densely populated rural areas, in which patients have to travel a long way to receive treatment. The necessity to travel to a more distant healthcare facility may result in patients and/or healthcare providers preferring to forego certain procedures. In addition, a variety of individual-level factors such as health literacy and language barriers could influence the type and amount of information delivered by health professionals and understood by the patient.

Future studies should investigate the factors contributing to inequalities to identify the best suited interventions. Structural drivers of disparities such as the availability of treatments or lack of trained healthcare professionals in hospitals serving lower SES areas can be addressed by purposeful relocation of resources to prevent and reduce inequalities. This may include strategic investments for more equitable roll-out of clinical innovation across territories, the establishment of ‘travelling’ expert clinics or teams to reduce travel barriers, or the introduction of patient navigators to help more vulnerable patients coordinate healthcare options and appointments.^51^ Individual-level contributing factors could be addressed by providing culturally and linguistically appropriate communication to women, so that they can make informed decisions about their care. This can be facilitated by appropriate training for healthcare professionals and the provision of adapted educational materials to facilitate provider–patient communication. Further research, supported by the multidisciplinary expertise of oncologists, epidemiologists, social scientists and social workers, among others, is necessary to understand and address socioeconomic disparities in breast cancer care.

## Supplementary material

10.1136/bmjqs-2024-017809 online supplemental file 1

10.1136/bmjqs-2024-017809 online supplemental file 2

## Data Availability

Data may be obtained from a third party and are not publicly available.
