# Supplementary material for: Socioeconomic inequalities in adherence to clinical practice guidelines and breast cancer survival: a multicentre population-based study in Spain
Source: BMJ Qual Saf. 2024 Dec 31;34(12):e017809. doi: 10.1136/bmjqs-2024-017809 (PMC12703246; doi:10.1136/bmjqs-2024-017809)

Petrova D, Redondo-Sánchez D, Rodríguez-Barranco M, et al. Socioeconomic inequalities in adherence to clinical practice guidelines and breast cancer survival: a multicentre population-based study in Spain. BMJ Qual Saf.
doi:10.1136/bmjqs-2024-017809

Figure S1. Map of the Spanish provinces contributing data to the European Cancer High Resolution Studies and socio-economic status (SES).

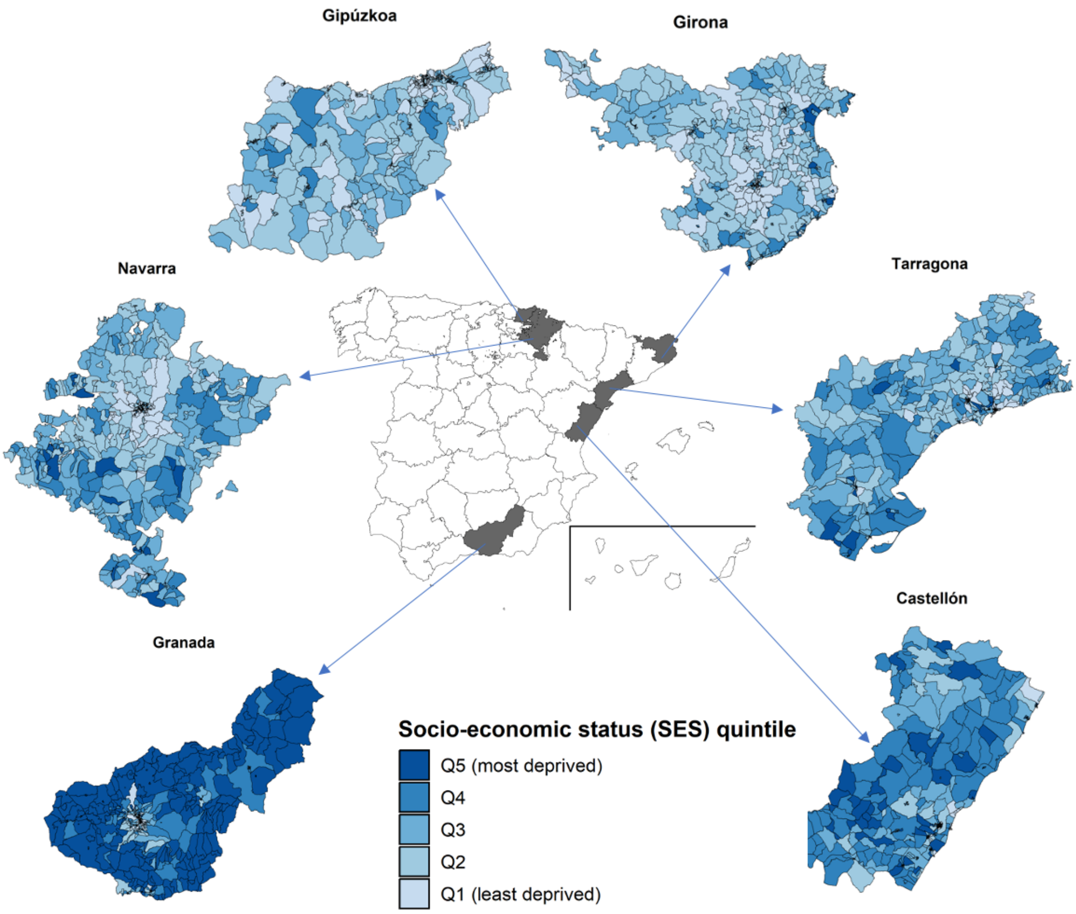

Supplement: online supplemental file 1 [file bmjqs-34-12-s001.pdf]
