# Supplementary material for: Socioeconomic inequalities in adherence to clinical practice guidelines and breast cancer survival: a multicentre population-based study in Spain
Source: BMJ Qual Saf. 2024 Dec 31;34(12):e017809. doi: 10.1136/bmjqs-2024-017809 (PMC12703246; doi:10.1136/bmjqs-2024-017809)
Supplement: online supplemental file 2 [file bmjqs-34-12-s002.pdf]

Table S1. Definition, period of introduction and completeness of indicators used to evaluate adherence to clinical practice guidelines for the diagnosis and treatment of breast cancer based on sources a, b, c, and d.

			Number of patients with missing data for the indicator / Number of patients to whom the indicator applied (Percentage)						
	**Indicator definition**	**Period of introduction in CPGs**	**Castellón (N = 559)**	**Gipuzkoa (N = 453)**	**Girona (N = 761)**	**Granada (N = 450)**	**Navarra (N = 441)**	**Tarragona (N = 542)**	**Overall (N = 3,206)**
Ind1	The patient's case was evaluated by the tumor-specific commission before starting treatment.	~2000s [1]	**	2/453 (0.4%)	163/761 (21.4%)	2/450 (0.4%)	12/441 (2.7%)	85/542 (15.7%)	264/2,647 (10.0%)
Ind2	The patient underwent full tumor classification (HER, ER, PR status).	~2005 [2]	0/559 (0%)	***	0/761 (0%)	0/450 (0%)	0/441 (0%)	0/542 (0%)	0/2,753 (0%)
Ind3	The patient underwent a sentinel lymph node biopsy (SLNB).	2000-2005 [3]	0/559 (0%)	5/453 (1.1%)	15/761 (2.0%)	2/450 (0.4%)	11/441 (2.5%)	17/542 (3.1%)	50/3,206 (1.6%)
Ind4	For patient with clinically negative axillary lymph nodes: The patient underwent SLNB.	2000-2005 [4]	0/41 (0%)	0/300 (0%)	2/464 (0.4%)	1/332 (0.3%)	5/52 (9.6%)	3/131 (2.3%)	11/1,320 (0.8%)
Ind5	For patients with a positive result in SLNB: The patient underwent axillary lymph node dissection.	2005-2010 [5]	0/104 (0%)	0/87 (0%)	0/155 (0%)	0/111 (0%)	1/111 (0.9%)	0/94 (0%)	1/662 (0.2%)
Ind6	The patient had 10 or more lymph nodes removed and examined during axillary lymph node dissection.	2000s [6]	287/429^ (66.9%)	4/158 (2.5%)	18/233 (7.7%)	1/233 (0.4%)	11/117 (9.4%)	21/274 (7.7%)	342/1,444 (23.7%)
Ind7	The patient was surgically treated with breast-conserving surgery (conservative treatment).	1990s [7]	0/559 (0%)	0/453 (0%)	0/761 (0%)	0/450 (0%)	0/441 (0%)	0/542 (0%)	0/3,206 (0%)
Ind8	For patients with stage I / IIA disease: The patient was treated with conservative surgery and receiving adjuvant radiotherapy.	2000s [8]	0/327 (0%)	0/282 (0%)	0/446 (0%)	0/303 (0%)	0/290 (0%)	0/275 (0%)	0/1,923 (0%)
Ind9	For patients undergoing mastectomy: The patient underwent reconstruction.	2000-2005 [9]	0/248 (0%)	2/100 (2.0%)	32/203 (15.8%)	7/95 (7.4%)	2/114 (1.8%)	13/128 (10.2%)	56/888 (6.3%)
Ind10	For patients with HER2+: The patient received targeted treatment.	2005-2006 [10]	1/88 (1.1%)	0/66 (0%)	2/84 (2.4%)	2/62 (3.2%)	2/64 (3.1%)	0/66 (0%)	7/430 (1.6%)
Ind11	For patients with HER2+: The patient received chemotherapy.	2005-2006 [11]	1/88 (1.1%)	0/66 (0%)	1/84 (1.2%)	1/62 (1.6%)	1/64 (1.6%)	0/66 (0%)	4/430 (0.9%)
Ind12	For patients with positive ER or PR receptors: The patient received hormonal treatment.	1990s [12]	3/467 (0.6%)	1/17 (5.9%)	8/652 (1.2%)	14/355 (3.9%)	4/361 (1.1%)	3/456 (0.7%)	33/2,308 (1.4%)
Ind13	For patients with triple negative cancer: The patient received chemotherapy.	2000s [13]	0/43 (0%)	0/6 (0%)	0/63 (0%)	0/44 (0%)	0/37 (0%)	0/44 (0%)	0/237 (0%)
Ind14	For patients with node-positive results: The patient underwent chemotherapy.	2000s [14]	3/266 (1.1%)	1/202 (0.5%)	2/352 (0.6%)	3/202 (1.5%)	1/186 (0.5%)	1/231 (0.4%)	11/1,439 (0.8%)
Ind15*	For patients in whom surgery is indicated: The patient underwent surgical intervention within 30 days after pathological diagnosis.	Beginning of 2000s [15]	0/502 (0%)	0/402 (0%)	4/671 (0.6%)	0/414 (0%)	1/416 (0.2%)	8/466 (1.7%)	13/2,871 (0.5%)
Ind16*	For patients who underwent surgery: The patient started adjuvant treatment within 6 weeks from the date of surgery.	Beginning of 2000s [16]	91/502 (18.1%)	51/402 (12.7%)	97/671 (14.5%)	65/414 (15.7%)	66/416 (15.9%)	71/466 (15.2%)	441/2,871 (15.4%)

* Number of days/weeks based on local guidelines of the Health Ministry of the Government of Andalucia, Spain (d).

** Castellón did not collect this information.

*** Gipuzkoa did not collect this information.

^ Castellón did not complete the data collection on this variable.

CPGs: Clinical practice guidelines; ER: Estrogen receptors; PR: Progesterone receptors; SLNB: sentinel lymph node biopsy

Sources:

(a) Allemani, C.; Storm, H.; Voogd, A.C.; Holli, K.; Izarzugaza, I.I.; Torrella-Ramos, A.; Bielska-Lasota, M.; Aareleid, T.; Ardanaz, E.; Colonna, M.; et al. Variation in “standard care” for breast cancer across Europe: A EUROCARE-3 high resolution study. Eur. J. Cancer 2010, 46, 1528–1536, doi:10.1016/j.ejca.2010.02.016.
(b) Aebi, S.; Davidson, T.; Gruber, G.; Cardoso, F.; ESMO Guidelines Working Group Primary breast cancer: ESMO Clinical Practice Guidelines for diagnosis, treatment and follow-up. Ann. Oncol. Off. J. Eur. Soc. Med. Oncol. 2011, 22 Suppl 6, vi12-24, doi:10.1093/annonc/mdr371.
(c) Del Barco, S.; Ciruelos, E.; Tusquets, I.; Ruiz, M.; Barnadas, A.; SEOM SEOM clinical guidelines for the systemic treatment of early breast cancer 2013. Clin. Transl. Oncol. 2013, 15, 1011–7, doi:10.1007/s12094-013-1084-3.
(d) Consejería de Salud; Junta de Andalucía Proceso Asistencial Integrado. Cáncer de Mama.; 2011; ISBN 84-8486-033-7.

Key scientific references supporting the recommendations:

[1] Junta de Andalucía. *Plan Integral de Oncología de Andalucía 2002-2005*. Sevilla: Consejería de Salud; 2002.https://www.juntadeandalucia.es/export/drupaljda/salud_5af0653399091_plan_oncologia_2002_2006.pdf
[2] Ministerio de Sanidad y Política Social. *Guía de Práctica Clínica en Cáncer de Mama*. Madrid: Ministerio de Sanidad y Política Social; 2009.https://www.sanidad.gob.es/areas/calidadAsistencial/excelenciaClinica/docs/Cancer_EyR.pdf
[3] Veronesi U, Paganelli G, Viale G, Luini A, Zurrida S, Galimberti V, et al. Sentinel lymph node biopsy and axillary dissection in breast cancer: Results in a large series. J Natl Cancer Inst. 1999;91(4):368-73.
[4] Veronesi U, Paganelli G, Viale G, Luini A, Zurrida S, Galimberti V, et al. A randomized comparison of sentinel-node biopsy with routine axillary dissection in breast cancer. N Engl J Med. 2003;349:546-53.
[5] Giuliano AE, McCall L, Beitsch P, Whitworth PW, Blumencranz PW, Leitch AM, et al. Sentinel lymph node dissection vs axillary dissection in patients with sentinel node metastases: A randomized clinical trial. JAMA. 2011;305(6):569-75.
[6] Goyal A, Newcombe RG, Chhabra A, Mansel RE. Factors affecting failed localisation and false-negative rates of sentinel node biopsy in breast cancer-results of the NEW START sentinel node biopsy training programme. Eur J Surg Oncol. 2006;32(5):499-504.
[7] Early Breast Cancer Trialists' Collaborative Group (EBCTCG). Effects of radiotherapy and surgery in early breast cancer: An overview of the randomized trials. N Engl J Med. 1995;333:1444-55.
[8] Fisher B, Anderson S, Bryant J, Margolese RG, Deutsch M, Fisher ER, et al. Twenty-year follow-up of a randomized trial comparing total mastectomy, lumpectomy, and lumpectomy plus irradiation for the treatment of invasive breast cancer. N Engl J Med. 2002;347:1233-41.
[9] Albornoz CR, Bach PB, Mehrara BJ, Disa JJ, Pusic AL, McCarthy CM, et al. A paradigm shift in U.S. breast reconstruction: Increasing implant rates. Plast Reconstr Surg. 2013;131(1):15-23.
[10] Romond EH, Perez EA, Bryant J, Suman VJ, Geyer CE, Davidson NE, et al. Trastuzumab plus adjuvant chemotherapy for operable HER2-positive breast cancer. N Engl J Med. 2005;353:1673-84.
[11] Slamon DJ, Leyland-Jones B, Shak S, Fuchs H, Paton V, Bajamonde A, et al. Use of chemotherapy plus a monoclonal antibody against HER2 for metastatic breast cancer that overexpresses HER2. N Engl J Med. 2001;344:783-92.
[12] Early Breast Cancer Trialists' Collaborative Group (EBCTCG). Tamoxifen for early breast cancer: An overview of the randomized trials. Lancet. 1998;351:1451-67.
[13] Carey LA, Dees EC, Sawyer L, Gatti L, Moore DT, Collichio F, et al. The triple negative paradox: Primary tumor chemosensitivity of breast cancer subtypes. Clin Cancer Res. 2007;13(8):2329-34.
[14] Mamounas EP, Anderson SJ, Dignam JJ, Bear HD, Julian TB, Geyer CE Jr, et al. Benefit from chemotherapy in breast cancer patients with 1-3 positive axillary nodes: Results from the randomized NSABP B-20 trial. J Clin Oncol. 2011;29(29):3366-73.
[15] American Society of Clinical Oncology (ASCO). Quality indicators for breast cancer care. J Clin Oncol. 2008;26:696-702.
[16] Lohrisch C, Paltiel C, Gelmon K, Speers C, Taylor S, Barnett J, et al. Impact on survival of time from definitive surgery to initiation of adjuvant chemotherapy for early-stage breast cancer. J Clin Oncol. 2006;24:4888-94.

Table S2. Adherence to the different clinical practice indicators as a function of demographic and clinical characteristics. P-values are from chi-square tests. *N= Number of patients to whom the indicator applied; N-adh = Number of patients with adherence on the indicator; % = percentage of patients with adherence: N-adh/N*.

		**Ind1**			**Ind2**			**Ind3**			**Ind4**			**Ind5**		
	**N***	2,647			2,753			3,206			1,320			662		
		**N-adh**	**N***	**%**	**N-adh**	**N***	**%**	**N-adh**	**N***	**%**	**N-adh**	**N***	**%**	**N-adh**	**N***	**%**
	**Overall**	2,228	2,647	84.2	2,589	2,753	94.0	1,966	3,206	61.3	1,000	1,320	75.8	512	662	77.3
**Province**	**Castellón**	--	--	--	531	559	95.0	272	559	48.7	7	41	17.1	87	104	83.7
	**Gipuzkoa**	443	453	97.8	--	--	--	299	453	66.0	265	300	88.3	60	87	69.0
	**Girona**	473	761	62.2	715	761	94.0	475	761	62.4	376	464	81.0	128	155	82.6
	**Granada**	446	450	99.1	420	450	93.3	296	450	65.8	245	332	73.8	97	111	87.4
	**Navarra**	425	441	96.4	419	441	95.0	311	441	70.5	7	52	13.5	59	111	53.2
	**Tarragona**	441	542	81.4	504	542	93.0	313	542	57.7	100	131	76.3	81	94	86.2
	p-value			***<0.001***			*0.537*			***<0.001***			***<0.001***			***<0.001***
**Age group**	**15-49**	630	727	86.7	749	785	95.4	607	899	67.5	298	346	86.1	190	241	78.8
	**50-69**	1,009	1,176	85.8	1,146	1,202	95.3	1,027	1,419	72.4	521	601	86.7	241	309	78.0
	**70-79**	344	418	82.3	410	427	96.0	260	490	53.1	145	218	66.5	66	89	74.2
	**80+**	245	326	75.2	284	339	83.8	72	398	18.1	36	155	23.2	15	23	65.2
	p-value			***<0.001***			***<0.001***			***<0.001***			***<0.001***			*0.395*
**Year**	**2010**	352	547	64.4	511	547	93.4	312	547	57.0	197	257	76.7	77	91	84.6
	**2011**	1,420	1,628	87.2	1,402	1,486	94.3	1,173	1,908	61.5	773	969	79.8	325	390	83.3
	**2012**	31	31	100.0	257	279	92.1	170	310	54.8	23	42	54.8	51	70	72.9
	**2013**	360	376	95.7	356	376	94.7	264	376	70.2	6	49	12.2	55	101	54.5
	**2014**	65	65	100.0	63	65	96.9	47	65	72.3	1	3	33.3	4	10	40.0
	p-value			***<0.001***			*0.446*			***<0.001***			***<0.001***			***<0.001***
**Comorbidity**	**0**	929	1,045	88.9	696	726	95.9	774	1,045	74.1	446	517	86.3	187	269	69.5
	**≥1**	1,074	1,216	88.3	1,015	1,084	93.6	701	1,216	57.6	384	552	69.6	182	227	80.2
	**Unknown**	225	386	58.3	878	943	93.1	491	945	52.0	170	251	67.7	143	166	86.1
	p-value			***<0.001***			***0.047***			***<0.001***			***<0.001***			***<0.001***

**Mode of detection**	**Symptomatic**	1,304	1,546	84.3	1,558	1,662	93.7	963	1,928	49.9	510	747	68.3	309	387	79.8
	**Screen-detected**	887	1,030	86.1	985	1,027	95.9	972	1,207	80.5	474	548	86.5	194	261	74.3
	**Unknown**	37	71	52.1	46	64	71.9	31	71	43.7	16	25	64.0	9	14	64.3
	p-value			*0.236*			***<0.001***			***<0.001***			***<0.001***			*0.145*
**Differentiation grade**	**1**	447	527	84.8	537	566	94.9	490	657	74.6	261	319	81.8	74	114	64.9
	**2**	908	1,055	86.1	1,051	1,092	96.2	881	1,306	67.5	437	549	79.6	270	348	77.6
	**3-4**	498	595	83.7	611	641	95.3	399	722	55.3	199	258	77.1	116	134	86.6
	**Unknown**	375	470	79.8	390	454	85.9	196	521	37.6	103	194	53.1	52	66	78.8
	p-value			0.262			**<0.001**			**<0.001**			**<0.001**			**0.001**
**Stage at diagnosis**	**I**	884	1,054	83.9	1,018	1,062	95.9	1,126	1,239	90.9	605	662	91.4	70	160	43.8
	**II**	747	858	87.1	887	923	96.1	663	1,086	61.0	344	493	69.8	311	355	87.6
	**III**	370	416	88.9	423	445	95.1	146	517	28.2	48	120	40.0	117	125	93.6
	**IV**	130	165	78.8	143	167	85.6	10	187	5.3	1	19	5.3	5	7	71.4
	**Unknown**	97	154	63.0	118	156	75.6	21	177	11.9	2	26	7.7	9	15	60.0
	p-value			***<0.001***			***<0.001***			***<0.001***			***<0.001***			***<0.001***

Petrova D, Redondo-Sánchez D, Rodríguez-Barranco M, et al. Socioeconomic inequalities in adherence to clinical practice guidelines and breast cancer survival: a multicentre population-based study in Spain. BMJ Qual Saf. doi:10.1136/bmjqs-2024-017809

		**Ind6**			**Ind7**			**Ind8**			**Ind9**			**Ind10**		
	**N***	1,444			3,206			1,923			888			430		
		**N-adh**	**N***	**%**	**N-adh**	**N***	**%**	**N-adh**	**N***	**%**	**N-adh**	**N***	**%**	**N-adh**	**N***	**%**
**Overall**		886	1,444	61.4	1,972	3206	61.5	1,500	1,923	78.0	282	888	31.8	348	430	80.9
**Province**	**Castellón**	112	429	26.1	254	559	45.4	198	327	60.6	68	248	27.4	75	88	85.2
	**Gipuzkoa**	139	158	88.0	301	453	66.4	231	282	81.9	40	100	40.0	58	66	87.9
	**Girona**	178	233	76.4	465	761	61.1	357	446	80.0	90	203	44.3	68	84	81.0
	**Granada**	157	233	67.4	318	450	70.7	244	303	80.5	10	95	10.5	48	62	77.4
	**Navarra**	101	117	86.3	298	441	67.6	236	290	81.4	35	114	30.7	46	64	71.9
	**Tarragona**	199	274	72.6	336	542	62.0	234	275	85.1	39	128	30.5	53	66	80.3
	p-value			***<0.001***			***<0.001***			***<0.001***			***<0.001***			*0.355*
**Age group**	**15-49**	292	456	64.0	559	899	62.2	414	540	76.7	185	305	60.7	160	172	93.0
	**50-69**	389	602	64.6	1,046	1,419	73.7	834	983	84.8	91	309	29.4	143	171	83.6
	**70-79**	154	239	64.4	271	490	55.3	198	271	73.1	6	163	3.7	36	55	65.5
	**80+**	51	147	34.7	96	398	24.1	54	129	41.9	0	111	0.0	9	32	28.1
	p-value			*0.931*			***<0.001***			***<0.001***			***<0.001***			***<0.001***
**Year**	**2010**	147	195	75.4	336	547	61.4	258	310	83.2	57	142	40.1	49	61	80.3
	**2011**	576	925	62.3	1,189	1,908	62.3	891	1,144	77.9	155	507	30.6	219	263	83.3
	**2012**	62	207	30.0	149	310	48.1	115	179	64.2	35	125	28.0	34	42	81.0
	**2013**	91	106	85.8	257	376	68.4	202	247	81.8	29	95	30.5	39	55	70.9
	**2014**	10	11	90.9	41	65	63.1	34	43	79.1	6	19	31.6	7	9	77.8
	p-value			***<0.001***			***<0.001***			***<0.001***			*0.088*			*0.331*
**Comorbidity**	**0**	297	367	80.9	744	1,045	71.2	586	699	83.8	115	249	46.2	139	160	86.9
	**≥1**	390	523	74.6	739	1,216	60.8	531	673	78.9	61	296	20.6	108	145	74.5
	**Unknown**	199	554	35.9	489	945	51.7	383	551	69.5	106	343	30.9	101	125	80.8
	p-value			***0.032***			***<0.001***			***<0.001***			***<0.001***			***0.015***
**Mode of detection**	**Symptomatic**	564	951	59.3	1,002	1,928	52.0	669	934	71.6	188	629	29.9	240	291	82.5
	**Screen-detected**	308	452	68.1	944	1,207	78.2	813	958	84.9	84	234	35.9	101	131	77.1
	**Unknown**	14	41	34.1	26	71	36.6	18	31	58.1	10	25	40.0	7	8	87.5
	p-value			*0.114*			***<0.001***			***<0.001***			***0.019***			*0.195*

**Differentiation grade**	**1**	119	233	51.1	483	657	73.5	432	518	83.4	42	137	30.7	15	23	65.2
	**2**	413	620	66.6	847	1,306	64.9	637	821	77.6	127	376	33.8	134	169	79.3
	**3-4**	244	379	64.4	433	722	60.0	284	364	78.0	64	237	27.0	146	170	85.9
	**Unknown**	110	212	51.9	209	521	40.1	147	220	66.8	49	138	35.5	53	68	77.9
	p-value			**0.006**			**<0.001**			**<0.001**			0.205			**0.039**
**Stage at diagnosis**	**I**	125	256	48.8	1,056	1,239	85.2	1054	1239	85.1	72	166	43.4	106	142	74.6
	**II**	460	678	67.8	656	1,086	60.4	446	684	65.2	124	369	33.6	125	146	85.6
	**III**	254	375	67.7	200	517	38.7	--	--	--	65	268	24.3	77	91	84.6
	**IV**	23	58	39.7	25	187	13.4	--	--	--	9	45	20.0	30	34	88.2
	**Unknown**	24	77	31.2	35	177	19.8	--	--	--	12	40	30.0	10	17	58.8
	p-value			***<0.001***			***<0.001***			***<0.001***			***0.002***			***0.005***

Petrova D, Redondo-Sánchez D, Rodríguez-Barranco M, et al. Socioeconomic inequalities in adherence to clinical practice guidelines and breast cancer survival: a multicentre population-based study in Spain. BMJ Qual Saf. doi:10.1136/bmjqs-2024-017809

		**Ind11**			**Ind12**			**Ind13**			**Ind14**			**Ind15**			**Ind16**		
	**N***	430			2,308			237			1,439			2,871			2,871		
		**N-adh**	**N***	**%**	**N-adh**	**N***	**%**	**N-adh**	**N***	**%**	**N-adh**	**N***	**%**	**N-adh**	**N***	**%**	**N-adh**	**N***	**%**
**Overall**		353	430	82.1	2,155	2,308	93.4	191	237	80.6	1,085	1,439	75.4	1,036	2,871	36.1	839	2,871	29.2
**Province**	**Castellón**	74	88	84.1	435	467	93.1	35	43	81.4	205	266	77.1	264	502	52.6	125	502	24.9
	**Gipuzkoa**	57	66	86.4	15	17	88.2	3	6	50.0	143	202	70.8	218	402	54.2	186	402	46.3
	**Girona**	69	84	82.1	617	652	94.6	52	63	82.5	253	352	71.9	290	671	43.2	152	671	22.7
	**Granada**	53	62	85.5	320	355	90.1	37	44	84.1	169	202	83.7	59	414	14.3	109	414	26.3
	**Navarra**	45	64	70.3	338	361	93.6	29	37	78.4	136	186	73.1	86	416	20.7	178	416	42.8
	**Tarragona**	55	66	83.3	430	456	94.3	35	44	79.5	179	231	77.5	119	466	25.5	89	466	19.1
	p-value			*0.194*			*0.701*			*0.514*			***0.008***			***<0.001***			***<0.001***
**Age group**	**15-49**	167	172	97.1	622	656	94.8	59	65	90.8	448	485	92.4	285	869	32.8	316	869	36.4
	**50-69**	149	171	87.1	976	1,036	94.2	89	97	91.8	521	590	88.3	521	1,359	38.3	446	1,359	32.8
	**70-79**	30	55	54.5	328	350	93.7	36	49	73.5	97	220	44.1	161	435	37.0	71	435	16.3
	**80+**	7	32	21.9	229	266	86.1	7	26	26.9	19	144	13.2	69	208	33.2	6	208	2.9
	p-value			***<0.001***			***<0.001***			***<0.001***			***<0.001***			***0.046***			***<0.001***
**Year**	**2010**	52	61	85.2	432	458	94.3	43	51	84.3	181	240	75.4	176	480	36.7	100	480	20.8
	**2011**	222	263	84.4	1,159	1,244	93.2	101	125	80.8	662	859	77.1	626	1,701	36.8	498	1,701	29.3
	**2012**	34	42	81.0	226	245	92.2	18	24	75.0	106	154	68.8	148	274	54.0	63	274	23.0
	**2013**	37	55	67.3	286	307	93.2	24	31	77.4	120	161	74.5	56	356	15.7	149	356	41.9
	**2014**	8	9	88.9	52	54	96.3	5	6	83.3	16	25	64.0	30	60	50.0	29	60	48.3
	p-value			*0.057*			*0.813*			*0.884*			*0.153*			***<0.001***			***<0.001***
**Comorbidity**	**0**	139	160	86.9	598	633	94.5	49	52	94.2	403	488	82.6	365	998	36.6	411	998	41.2
	**≥1**	111	145	76.6	829	891	93.0	77	109	70.6	360	528	68.2	265	1,037	25.6	241	1,037	23.2
	**Unknown**	103	125	82.4	728	784	92.9	65	76	85.5	322	423	76.1	406	836	48.6	187	836	22.4
	p-value			***0.042***			*0.689*			***<0.001***			***<0.001***			***<0.001***			***<0.001***
**Mode of detection**	**Symptomatic**	242	291	83.2	1,245	1,348	92.4	134	170	78.8	721	1,003	71.9	547	1,634	33.5	442	1,634	27.1
	**Screen-detected**	105	131	80.2	867	912	95.1	55	64	85.9	342	411	83.2	467	1,180	39.6	389	1,180	33.0
	**Unknown**	6	8	75.0	43	48	89.6	2	3	66.7	22	25	88.0	22	57	38.6	8	57	14.0
	p-value			*0.742*			***0.017***			*0.390*			***<0.001***			***0.002***			*0.144*

**Differentiation grade**	**1**	15	23	65.2	522	547	95.4	4	5	80.0	126	194	64.9	250	623	40.1	128	623	20.5
	**2**	136	169	80.5	939	989	94.9	38	54	70.4	478	629	76.0	468	1,225	38.2	398	1,225	32.5
	**3-4**	147	170	86.5	376	416	90.4	128	147	87.1	319	382	83.5	224	672	33.3	260	672	38.7
	**Unknown**	55	68	80.9	318	356	89.3	21	31	67.7	162	234	69.2	94	351	26.8	53	351	15.1
	p-value			**0.045**			**<0.001**			**0.013**			**<0.001**			**<0.001**			**<0.001**
**Stage at diagnosis**	**I**	105	142	73.9	898	948	94.7	45	58	77.6	115	182	63.2	546	1,224	44.4	331	1,224	27.0
	**II**	130	146	89.0	733	770	95.2	87	99	87.9	500	634	78.9	329	1,029	32.0	336	1,029	32.7
	**III**	77	91	84.6	321	345	93.0	45	59	76.3	351	445	78.9	127	468	27.1	155	468	33.1
	**IV**	31	34	91.2	116	138	84.1	6	9	66.7	89	124	71.8	16	71	22.5	7	71	9.9
	**Unknown**	10	17	58.8	87	107	81.3	8	12	66.7	30	54	55.6	18	79	22.8	10	79	12.7
	p-value			***<0.001***			***<0.001***			*0.136*			***<0.001***			***<0.001***			***<0.001***

Table S3. Number of patients at risk (N) and observed survival (OS) at 1, 3 and 5 years as a function of socio-economic status (SES) quintile.

		N	1-year OS (95% CI)	3-years OS (95% CI)	5-years OS (95% CI)
**Overall**		3,206	96.1 (95.3-96.7)	89.0 (87.9-90.1)	83.5 (82.2-84.7)
**Socio-economic status (SES) quintile**	**Q1 (highest SES)**	665	96.8 (95.2-97.9)	91.1 (88.7-93.1)	85.6 (82.7-88.0)
	**Q2**	912	96.5 (95.1-97.5)	87.6 (85.3-89.6)	82.9 (80.3-85.2)
	**Q3**	813	96.6 (95.1-97.6)	91.3 (89.1-93.0)	85.2 (82.6-87.5)
	**Q4**	510	95.1 (92.8-96.7)	88.2 (85.1-90.7)	82.1 (78.5-85.2)
	**Q5 (lowest SES)**	306	93.5 (90.1-95.7)	83.9 (79.4-87.7)	78.4 (73.4-82.6)

Table S4. Number of patients at risk (N) and marginal relative survival (MRS) at 1, 3 and 5 years as a function of socio-economic status (SES) quintile, adjusted for age and year of diagnosis.

		N	1-year MRS (95% CI)	3-years MRS (95% CI)	5-years MRS (95% CI)
**Overall**		3,206	97.7 (97.2-98.3)	94.7 (93.8-95.6)	92.3 (91.1-93.6)
**Socio-economic status (SES) quintile**	**Q1 (highest SES)**	665	97.8 (96.9-98.7)	94.8 (93.0-96.6)	92.4 (89.9-95.0)
	**Q2**	912	97.5 (96.7-98.3)	94.2 (92.7-95.8)	91.6 (89.4-93.9)
	**Q3**	813	98.8 (98.2-99.3)	97.1 (95.8-98.3)	95.7 (93.9-97.5)
	**Q4**	510	97.2 (96.1-98.4)	93.6 (91.4-95.9)	90.7 (87.6-94.0)
	**Q5 (lowest SES)**	306	96.4 (94.9-98.0)	91.7 (88.6-94.9)	88.0 (83.7-92.5)

Table S5. Effects of socio-economic status (SES) and adherence to each indicator derived from flexible parametric models of relative survival adjusted for age, year of diagnosis, immunohistochemical profile, grade of differentiation, and stage at diagnosis. *Note:* N=Number of patients at risk (to which the adherence indicator applies and is not unknown); HR=mortality hazard ratios (HR) and 95% confidence interval (CI). No results are presented for Ind8 because of lack of convergence.

		**HR (95% CI)**	
	**N**	**SES Q4-Q5 vs. Q1**	**Adherence vs. non-adherence**
**Ind1**	2,383	1.21 (0.78-1.90)	0.80 (0.44-1.47)
**Ind2**	2,753	1.49 (0.95-2.32)	1.07 (0.47-2.44)
**Ind3**	3,156	1.36 (0.91-2.04)	**0.20 (0.11-0.36)**
**Ind4**	1,309	0.58 (0.22-1.53)	**0.17 (0.05-0.57)**
**Ind5**	661	1.85 (0.28-12.31)*	1.34 (0.30-6.03)*
**Ind6**	1,102	1.54 (0.62-3.84)	0.61 (0.30-1.25)
**Ind7**	3,206	**1.54 (1.03-2.32)**	**0.32 (0.22-0.47)**
**Ind8**	1,923	-	-
**Ind9**	832	2.25 (0.80-6.32)	**0.28 (0.13-0.60)**
**Ind10**	423	3.19 (0.79-12.85)	0.68 (0.22-2.12)
**Ind11**	426	2.94 (0.79-10.89)	0.74 (0.21-2.55)
**Ind12**	2,275	0.92 (0.53-1.60)	**0.11 (0.06-0.17)**
**Ind13**	237	0.87 (0.35-2.19)	**0.43 (0.18-1.04)**
**Ind14**	1,428	1.29 (0.80-2.10)	**0.59 (0.36-0.97)**
**Ind15**	2,858	1.27 (0.71-2.29)	0.76 (0.45-1.27)
**Ind16**	2,430	0.77 (0.39-1.52)	**0.54 (0.29-0.99)**

* HR not adjusted by stage because of lack of convergence.
